# Supplementary material for: Correlation detection as a stimulus computable account for audiovisual perception, causal inference, and saliency maps in mammals
Source: eLife. 2025 Nov 4;14:RP106122. doi: 10.7554/eLife.106122 (PMC12585176; doi:10.7554/eLife.106122)
Supplement: Supplementary file 1. — The first column contains the reference of the study, the second column the task (McGurk, Simultaneity Judgment, and Temporal Order Judgment). The third column describes the stimuli: n represents the number of individual instances of the stimuli, ‘HI’ and ‘LI’ in Magnotti and Beauchamp, 2017 indicate speech stimuli with High and Low Intelligibility, respectively. ‘Blur’ indicates that the videos were blurred. ‘Disamb’ indicates that ambiguous speech stimuli (i.e., sine-wave speech) were disambiguated by informing the observers of the original speech sound. The fourth column indicates whether visual and acoustic stimuli were congruent. Here, incongruent stimuli refer to the mismatching speech stimuli used in the McGurk task. ‘SWS’ indicates sine-wave speech; ‘noise’ in Ikeda and Morishita, 2020 indicates a stimulus similar to sine-wave speech but in which white noise was used instead of pure sinusoidal waves. The fifth column represents the country where the study was performed. The sixth column describes the observers included in the study: ‘c.s.’ indicates convenience sampling (usually undergraduate students) musicians in Lee and Noppeney, 2011; Lee and Noppeney, 2014 were amateur piano players; Freeman et al., 2013 tested young observers (18–28 years old), a patient P.H. (67 years old) that after a lesion in the pons and basal ganglia reported hearing speech before seeing the lips move; and a group of age-matched controls (59–74 years old). The seventh column reports the number of observers included in the study. Overall, the full dataset consisted of 986 individual psychometric curves; however, several observers participated in more than one experiment, so that the total number of unique observers was 454. The eight column reports the number of lags used in the method of constant stimuli. The nineth column reports the number of trials included in the study. The tenth column reports the correlation between empirical and predicted psychometric functions. The bottom [file elife-106122-supp1.docx]

| **Reference** | **Task** | **Stimuli** | **Congr.** | **Country** | **Observers** | **Num. obser.** | **Num lag** | **Num. trials** | **MCD-**  **data correl.** |
| --- | --- | --- | --- | --- | --- | --- | --- | --- | --- |
| Van Wassenhove et al. (2007) | McGurk | [V,A]=[/ga/,/ba/] | no | US | c.s. | 18 | 29 | 5,580 | .933 |
| Van Wassenhove et al. (2007) | McGurk | [V,A]=[/ka/,/pa/] | no | US | c.s. | 21 | 29 | 6,510 | .983 |
| Freeman et al. (2013) | McGurk | Syllables (n=3) | no | UK | young | 27 | 9 | 4,374 | .95 |
| Freeman et al. (2013) | McGurk | Syllables (n=3) | no | UK | elderly | 10 | 9 | 1,620 | .939 |
| Freeman et al. (2013) | McGurk | Syllables (n=3) | no | UK | Patient P.H. | 1 | 9 | 162 | .905 |
| Yuan et al. (2014) | McGurk | [V,A]=[/ga/,/ba/] | no | CN | c.s. | 12 | 9 | 1,080 | .905 |
| Van Wassenhove et al. (2007) | SJ | [V,A]=[/ga/,/ba/] | no | US | c.s. | 18 | 29 | 3,132 | .962 |
| Van Wassenhove et al. (2007) | SJ | [V,A]=[/ka/,/pa/] | no | US | c.s. | 21 | 29 | 3,654 | .968 |
| Van Wassenhove et al. (2007) | SJ | /da/ | yes | US | c.s. | 18 | 29 | 3,132 | .983 |
| Van Wassenhove et al. (2007) | SJ | /ta/ | yes | US | c.s. | 21 | 29 | 3,654 | .989 |
| Roseboom and Arnold (2011) | SJ | /ba/ (male/female) | yes | UK | c.s. | 6 | 9 | 1,296 | .974 |
| Yuan et al. (2014) | SJ | [V,A]=[/ga/,/ba/] | no | CN | c.s. | 13 | 9 | 1,170 | .991 |
| Lee and Noppeney (2011) | SJ | Sentences (n=16) | yes | DE | c.s. | 19 | 13 | 15,808 | .991 |
| Lee and Noppeney (2011) | SJ | Sentences ( n=16) | yes | DE | musicians | 18 | 13 | 14,976 | .99 |
| Lee and Noppeney (2014) | SJ | Sentences (n=16) | sws | DE | c.s.+musicians | 37 | 13 | 30,784 | .985 |
| Lee and Noppeney (2011) | SJ | Piano melodies (n=16) | yes | DE | c.s. | 19 | 13 | 15,808 | .969 |
| Lee and Noppeney (2011) | SJ | Piano melodies (n=16) | yes | DE | musicians | 18 | 13 | 14,976 | .992 |
| Ikeda and Morishita (2020) | SJ | Sentences (n=4) | yes | JP | c.s. | 28 | 13 | 4,368 | .986 |
| Ikeda and Morishita (2020) | SJ | Sentences (n=6) | yes | JP | c.s. | 22 | 13 | 3,432 | .995 |
| Ikeda and Morishita (2020) | SJ | Sentences (n=6) | noise | JP | c.s. | 22 | 13 | 3,432 | .979 |
| Ikeda and Morishita (2020) | SJ | Flute melodies (n=4) | yes | JP | c.s. | 28 | 13 | 4,368 | .95 |
| Ikeda and Morishita (2020) | SJ | Piano melodies (n=4) | yes | JP | c.s. | 28 | 13 | 4,368 | .93 |
| Magnotti and Beauchamp (2013) | SJ | Words (n=4) HI | yes | US | c.s. | 39 | 15 | 13,320 | .989 |
| Magnotti and Beauchamp (2013) | SJ | Words (n=4) HI, blur | yes | US | c.s. | 39 | 15 | 13,320 | .995 |
| Magnotti and Beauchamp (2013) | SJ | Words (n=4) LI | yes | US | c.s. | 39 | 15 | 13,320 | .99 |
| Magnotti and Beauchamp (2013) | SJ | Words (n=4) LI, blur | yes | US | c.s. | 39 | 15 | 13,320 | .991 |
| Van Laarhoven et al. (2019) | SJ | /tabi/ | yes | NL | c.s. | 101 | 21 | 31,815 | .99 |
| Vroomen et al. (2011) | SJ | /tabi/ | yes | NL | c.s. | 30 | 17 | 12,240 | .996 |
| Vroomen et al. (2011) | SJ | /tabi/ | sws | NL | c.s. | 30 | 17 | 12,240 | .992 |
| Vroomen et al. (2011) | SJ | /tabi/ (disamb.) | sws | NL | c.s. | 30 | 17 | 12,240 | .989 |
| Freeman et al. (2013) | TOJ | Syllables (n=5) | no | UK | young | 27 | 9 | 8,740 | .99 |
| Freeman et al. (2013) | TOJ | Syllables (n=5) | no | UK | elderly | 10 | 9 | 3,240 | .996 |
| Freeman et al. (2013) | TOJ | Syllables (n=5) | no | UK | Patient P.H. | 1 | 9 | 324 | .992 |
| Vroomen et al. (2011) | TOJ | /tabi/ | yes | NL | c.s. | 30 | 17 | 12,240 | .998 |
| Vroomen et al. (2011) | TOJ | /tabi/ | sws | NL | c.s. | 30 | 17 | 12,240 | .997 |
| Vroomen et al. (2011) | TOJ | /tabi/ (disamb.) | sws | NL | c.s. | 30 | 17 | 12,240 | .997 |
| Petrini et al. (2020) | TOJ | /tomorrow/ | Yes | UK | 7-8 years old | 15 | 7 | 1,050 | .958 |
| Petrini et al. (2020) | TOJ | /tomorrow/ | Yes | UK | 10-11 years old | 14 | 7 | 980 | .972 |
| Petrini et al. (2020) | TOJ | /tomorrow/ | Yes | UK | Adults | 14 | 7 | 980 | .988 |
| Petrini et al. (2020) | SJ | /tomorrow/ | Yes | UK | 7-8 years old | 15 | 7 | 1,050 | .994 |
| Petrini et al. (2020) | SJ | /tomorrow/ | Yes | UK | 10-11 years old | 14 | 7 | 980 | .980 |
| Petrini et al. (2020) | SJ | /tomorrow/ | Yes | UK | Adults | 14 | 7 | 980 | .990 |
|  |  | **105 unique stimuli** |  | **6 unique** |  | **986** |  | **324,543** | **0.981** |
|  |  |  |  | **countries** |  | **(individual** |  | **(total)** | **(mean)** |
|  |  |  |  |  |  | **curves)** |  |  |  |
|  |  |  |  |  |  | **454 (unique** |  |  |  |
|  |  |  |  |  |  | **observers)** |  |  |  |

**Supplementary File 1. Summary table of the experiments simulated in Figure 2-figure supplement 1.** The **first column** contains the reference of the study, the **second column** the task (McGurk, Simultaneity Judgment, and Temporal Order Judgment). The **third column** describes the stimuli: n represents the number of individual instances of the stimuli, “HI” and “LI” in Magnotti and Beauchamp (2013) indicate speech stimuli with High and Low Intelligibility, respectively. “Blur” indicates that the videos were blurred. “Disamb” indicates that ambiguous speech stimuli (i.e., sine-wave speech) were disambiguated by informing the observers of the original speech sound. The **fourth column** indicates whether visual and acoustic stimuli were congruent. Here, incongruent stimuli refer to the mismatching speech stimuli used in the McGurk task. “SWS” indicates sine-wave speech; “noise” in Ikeda and Morishita (2020) indicates a stimulus similar to sine-wave speech but in which white noise was used instead of pure sinusoidal waves. The **fifth column** represents the country where the study was performed. The **sixth column** describes the observers included in the study: “c.s.” indicates convenience sampling (usually undergraduate students) musicians in Lee and Noppeney (2011, 2014) were amateur piano players; Freeman et al. (2013) tested young observers (18-28 years old), a patient P.H. (67 years old) that after a lesion in the pons and basal ganglia reported hearing speech before seeing the lips move; and a group of age matched controls (59-74 years old). The **seventh column** reports the number of observers included in the study. Overall, the full dataset consisted of 986 individual psychometric curves; however, several observers participated in more than one experiment, so that the total number of unique observers was 454. The **eight column** reports the number of lags used in the method of constant stimuli. The **nineth column** reports the number of trials included in the study. The **tenth column** reports the correlation between empirical and predicted psychometric functions. The bottom row contains some descriptive statistics of the dataset.
