# Supplementary material for: Correlation detection as a stimulus computable account for audiovisual perception, causal inference, and saliency maps in mammals
Source: eLife. 2025 Nov 4;14:RP106122. doi: 10.7554/eLife.106122 (PMC12585176; doi:10.7554/eLife.106122)
Supplement: Supplementary file 2. — The first column contains the reference of the study, the second column the task (Simultaneity Judgment and Temporal Order Judgment). The third column describes the stimuli. The fourth column indicates what rats were used as observers. The fifth column reports the number of rats in the study; ‘same’ means that the same rats took part in the experiment in the row above. The sixth column reports the number of lags used in the method of constant stimuli. The seventh column reports the number of trials included in the study (not available for all studies). The eighth column reports the correlation between empirical and predicted psychometric functions. The bottom row contains some descriptive statistics of the dataset. [file elife-106122-supp2.docx]

| **Reference** | **Task** | **Stimuli** | **Observers** | **Num. obser.** | **Num lag** | **Num. trials** | **MCD-data**  **correl.** |
| --- | --- | --- | --- | --- | --- | --- | --- |
| Mafi et al. (2023) | TOJ | Click/flash | Male Wistar rats | 8 | 14 | 28,784 | .996 |
| Mafi et al. (2023) | TOJ | Click/flash | Humans | 10 | 14 | 12,964 | .992 |
| Schormans et al. (2017) | SJ | Click/flash | Male rats | 7 | 5 | 7,000 | .999 |
| Schormans et al. (2017) | TOJ | Click/flash | Male rats | 7 | 7 | 7,000 | .994 |
| Paulcan et al. (2023) | TOJ | Click/flash | male Sprague–Dawley rats | 8 | 8 | 2,880 | .996 |
| Paulcan et al. (2023) | TOJ | Click/flash | male Sprague–Dawley rats | 13 | 8 | 4,680 | .991 |
| Al-youzbaki et al. (2023) | TOJ | Click/flash | Sprague Dawley rats | 10 | 9 | 32,400 | .996 |
| Al-youzbaki et al. (2023) | SJ | Click/flash | Sprague Dawley rats | 12 | 11 | 47,520 | .983 |
| Al-youzbaki et al. (2023) | TOJ | Click/flash | Sprague Dawley rats / saline | 10 | 9 | 32,400 | .997 |
| Al-youzbaki et al. (2023) | TOJ | Click/flash | Sprague Dawley rats / MK-801 | 10 (same) | 9 | 32,400 | .999 |
| Al-youzbaki et al. (2023) | SJ | Click/flash | Sprague Dawley rats / saline | 8 | 11 | 31,680 | .996 |
| Al-youzbaki et al. (2023) | SJ | Click/flash | Sprague Dawley rats / MK-801 | 8 (same) | 11 | 31,680 | .991 |
| Schormans & Allman (2018) | TOJ | Click/flash  Loudness | Sprague Dawley rats | 10 | 7 | n/a | .989 |
| Schormans & Allman (2018) | SJ | Click/flash  Loudness | Sprague Dawley rats | 10 | 5 | n/a | .996 |
| Schormans & Allman (2023) | TOJ | Click/flash | Sprague Dawley rats | 8 | 7 | n/a | .995 |
| Schormans & Allman (2023) | TOJ | Click/flash | Sprague Dawley rats / gabazine | 8 (same) | 7 | n/a | .99 |
|  |  |  |  | **193**  **(individual curves)**  **110 (rats)** |  | **271,388(total)** | **.994**  **(mean)** |

**Supplementary Table 2. Summary of the experiments simulated in Figure 2-figure supplement 2.** The **first column** contains the reference of the study, the **second column** the task (Simultaneity Judgment, and Temporal Order Judgment). The **third column** describes the stimuli. The **fourth column** indicates what rats were used as observers. The **fifth column** reports the number of rats in the study; ‘same’ means that the same rats took part in the experiment in the row above. The **sixth column** reports the number of lags used in the method of constant stimuli. The **seventh column** reports the number of trials included in the study (not available for all studies). The **eight column** reports the correlation between empirical and predicted psychometric functions. The bottom row contains some descriptive statistics of the dataset.
